# Patient-derived d-MMR/MSI phenotype urachal cancer organoids for personalized drug screening

**DOI:** 10.3389/fonc.2026.1773072

**Published:** 2026-03-05

**Authors:** Kuangen Zhang, Xinyi Li, Zhenting Zhang, Ning Zhang, Xin Yao, Rong Liu

**Affiliations:** 1 Translational Cancer Research Center, First Hospital, Peking University, Beijing, China; 2 Department of Immunology, Peking University Health Science Center, Beijing, China; 3 Department of Genitourinary Oncology, Tianjin Medical University Cancer Institute and Hospital, Tianjin, China

**Keywords:** d-MMR/MSI phenotype, drug screening, organoid, personalized medicine, urachal cancer

## Abstract

**Background:**

Urachal cancer (UrC) is a rare, aggressive malignancy typically diagnosed at advanced stages, where systemic treatment becomes necessary. However, cytotoxic chemotherapy offers limited efficacy, and prospective clinical trials are exceedingly difficult due to the rarity of the disease. Thus, robust *in vitro* models are urgently needed to support precision medicine approaches for UrC.

**Methods:**

Fresh UrC tumor samples were collected from patients undergoing en bloc resection and cultured to generate PDOs. These organoids were subjected to drug screening using standard chemotherapeutic agents. Whole-exome sequencing (WES) and RNA sequencing (RNA-seq) were conducted to compare the molecular profiles of the PDOs with their corresponding parental tumors. Associations between drug responses and genomic/transcriptomic features were analyzed. Student’s *t*-test was used for statistical assessment.

**Results:**

The established d-MMR/MSI phenotype UrC PDOs faithfully reproduced the genomic and transcriptomic landscapes of the original tumors, including intratumoral heterogeneity, and demonstrated consistent drug response profiles. Molecular characterization further revealed actionable targets within the RAS/MAPK and PI3K/AKT/mTOR pathways, as well as immune-related targets such as PD-L1. These findings highlight the utility of PDOs in modeling rare cancers and guiding personalized therapeutic strategies.

**Conclusions:**

d-MMR/MSI phenotype UrC PDOs recapitulate the phenotypic and molecular features of their parental tumors, capturing critical heterogeneity. As such, they represent a valuable platform for reflecting treatment responses, investigating resistance mechanisms, and developing individualized therapeutic regimens.

## Introduction

Urachal cancer (UrC) is a rare and aggressive tumor originating from the urachus, a vestigial structure connecting the fetal allantois to the bladder dome (1). It accounts for less than 1% of all bladder cancers (2, 3), with over 90% of cases presenting as adenocarcinomas that share histopathological features with both primary bladder adenocarcinoma and colorectal adenocarcinoma (4). In localized disease, the standard of care involves partial cystectomy with en bloc resection of the urachal ligament and umbilicus (5). However, for recurrent or metastatic disease, systemic therapies—such as 5-fluorouracil-based regimens, cisplatin combinations, or hyperthermic intraperitoneal chemotherapy—are employed with limited efficacy (6–8). Radiotherapy remains largely ineffective for UrC, and outcomes for advanced disease are poor. Although the rarity of the tumor has historically meant an absence of standardized chemotherapy protocols, it is important to note that the prospective clinical trial in UrC patients has now been published (9). This emerging evidence, along with data from ongoing studies (10), directly addresses the previous lack of clinical trials and will be instrumental in guiding treatment decisions. However, the lack of consensus treatment guidelines still underscores the urgent need for robust preclinical models to facilitate research and inform therapeutic decision-making.

Patient-derived organoids (PDOs) have emerged as promising preclinical tools capable of modeling tumor heterogeneity and enabling individualized drug screening across multiple cancer types (11–14). Notably, PDOs preserve the genomic landscape and histological architecture of the primary tumor while providing predictive insight into treatment responses (15), highlighting their potential in personalized oncology. This is particularly crucial for rare malignancies, where low incidence rates and ethical constraints limit the feasibility of clinical trials. Organoid technology thus offers a powerful solution by enabling high-fidelity modeling of tumor biology and patient-specific drug responses *in vitro*.

In this study, we established four PDO lines from a single deficient Mismatch Repair/Microsatellite Instability (d-MMR/MSI) UrC patient and demonstrated their capacity to replicate the phenotypic and genomic heterogeneity of the corresponding tumors. These PDOs exhibited strong concordance with clinical responses to 5-fluorouracil (5-FU) and cisplatin, two standard chemotherapeutic agents. Furthermore, based on insights from whole-exome and transcriptomic profiling, we evaluated several FDA recently approved or investigational compounds and identified potential therapeutic candidates targeting the RAS/MAPK pathway, the PI3K/AKT/mTOR signaling pathway, and immune checkpoints in UrC organoids, and found that this d-MMR/MSI UrC patient is potentially sensitive to these inhibitors’ treatment. Collectively, our findings provide compelling evidence that UrC PDOs can serve as a translational platform for precision oncology, supporting their use in personalized treatment development and research for rare cancers.

## Materials and methods

### Human tissue collection

Fresh tumor and matched normal tissues from patients diagnosed with urachal carcinoma (UrC) or bladder cancer (BC) were obtained from the Department of Urology at Tianjin Medical University Cancer Institute and Hospital. Detailed bladder cancer organoids clinicopathological data are summarized in **Supplementary Table 1**. All procedures were approved by the Peking University First Hospital and Tianjin Medical University Cancer Institute review board (approval number: 2023051-001), and written informed consent was obtained from each participant. All research was performed in accordance with relevant guidelines/regulations. Research involving human participants has been conducted in accordance with the Declaration of Helsinki.

### Establishment of UrC and BC organoids

Tumor specimens were transported on ice to the laboratory and immediately processed. Samples were collected by the pathologist immediately following surgical resection. Resected tissues were minced and enzymatically digested using Collagenase I (1 mg/mL, Worthington), Collagenase II (1 mg/mL, Worthington), and Hyaluronidase (100 U/mL, Sigma-Aldrich) at 37 °C for 30–60 minutes. The tissue suspension was further digested in TrypLE Express (Invitrogen) at 37 °C for 10 minutes. Following filtration and washing, cells were resuspended in growth factor–reduced Matrigel (Corning), seeded into 6-well plates (Thermo Fisher Scientific), and overlaid with culture medium. The organoid medium, adapted from previously reported protocols for BC organoids (16), consisted of Advanced DMEM/F12 supplemented with 1× HEPES, 1× GlutaMax (Invitrogen), 1× Primocin (InvivoGen), 1× B27 Supplement (Gibco), 0.5 μM A83-01 (Selleck), 10 μM Y27632 (Selleck), 0.1 μg/mL FGF-10 (Novoprotein), 1.25 mM N-acetylcysteine (Sigma-Aldrich), and 12.5 mM nicotinamide (Sigma-Aldrich). Medium was refreshed every 2–3 days. For passaging, organoids were first released from Matrigel using 1 mg/mL dispase (STEMCELL Technologies) for 60 minutes at 37 °C, then dissociated into single cells with TrypLE Express. For cryopreservation, organoids were dissociated into single cells or small clusters and frozen in cryopreservation medium (Corning).

### Histology and immunohistochemistry

Organoids and tumor tissues were fixed in 4% paraformaldehyde, embedded in paraffin, sectioned, and stained with hematoxylin and eosin (H&E). Immunohistochemistry (IHC) was performed using the Roche Ventana Discovery Ultra automated system after heat-induced antigen retrieval. Primary antibodies used were: anti-MLH1 (1:100, ZM-0152), anti-PMS2 (1:100, ZA-0542), anti-MSH2 (1:100, ZA-0622), anti-MSH6 (1:100, ZA-0541), and anti-CK20 (1:100, ZA-0574), all obtained from Zhongshan Golden Bridge Biotechnology (Beijing, China). Two board-certified pathologists independently confirmed all histopathological evaluations in accordance with standard diagnostic criteria (17).

### Organoid drug screening

Organoids were enzymatically dissociated and passed through a 70 μm strainer (Corning) to remove large aggregates. The resulting suspension (20,000–50,000 organoids/mL in 5% Matrigel-containing medium) was plated in ultralow-attachment 384-well plates (Corning) in triplicate. After 24 hours, a dilution series of each drug was added. Concentration ranges varied depending on compound properties: 100 μM to 12.5 μM, 100 μM to 0.78 μM, or 20 μM to 2.5 μM (maximum DMSO concentration: 1%). After 6 days of incubation, cell viability was assessed using CellTiter-Glo 3D (Promega), following the manufacturer’s instructions. Data were normalized to vehicle controls, and IC_50_ and AUC values were calculated using nonlinear regression (log[inhibitor] *vs*. normalized response, variable slope) in GraphPad Prism 10.0b.

### Immune stimulation assays in organoids

Organoids and peripheral blood mononuclear cells (PBMCs) were co-cultured at a 1:5 ratio in medium supplemented with 100 U/mL IL-2, 10 ng/mL IL-7, and 10 ng/mL IL-15 (all from Peprotech). PBMCs were autologous (derived from the same patient) to ensure the validity of the immune co-culture assays. A total of 500 organoids and 2,500 PBMCs were seeded per well in 384-well plates and treated with either 20 μg/mL atezolizumab (Selleck) or IgG1 isotype control. After 72 hours, cell viability was measured using CellTiter-Glo 3D.

### Quantitative real-time PCR

Total RNA was extracted using the TRIzol reagent and reverse-transcribed to cDNA with a commercial RT-PCR kit (TianGen, Beijing, China). Quantitative PCR was performed on an AriaMx Real-Time PCR System (Agilent Technologies) using SYBR Green (TianGen) according to the manufacturer’s protocol. Primer sequences are listed in **Supplementary Table 2**.

### siRNA transfection

Silencer Select predesigned siRNAs targeting PCSK1 (GM-SI-147903) and CLUL1 (GM-SI-147979), as well as a non-targeting negative control siRNA, were purchased from Genomeditech (Shanghai, China). Organoids were dissociated into single cells and transfected using Lipofectamine RNAiMAX (Invitrogen), following minor modifications to the manufacturer’s protocol. Following siRNA transfection, cells were subjected to drug treatment for 6 days, and viability was assessed using CellTiter-Glo 3D.

### RNA sequencing and quantification

Total RNA was isolated by using the DNeasy & RNeasy kit (Qiagen), and poly-T oligo-attached magnetic beads were used for mRNA purification. Sequencing libraries were prepared and sequenced on the Illumina NovaSeq 6000 platform, generating 150 bp paired-end reads. Quality control of raw reads was performed with FastQC (v0.11.9), Cutadapt (v2.5, using Illumina universal adapters), and Trimmomatic (v0.39, parameters: PE, MINLEN = 36). Reads were aligned to the human reference genome (hg19) using STAR (v2.7.3a) with default settings. Gene-level counts were generated using HTSeq-count with GENCODE annotations, and transcript per million (TPM) values were calculated using RSEM. Unless otherwise stated, mRNA expression is presented as log_2_(TPM + 1). Correlation heatmaps comparing organoids and matched tumor tissues were generated as previously described (12), and statistical significance for expression variance across samples was assessed using one-way ANOVA.

RNA were extracted from fresh-frozen tissues and passage 1 organoids. We also confirmed that histological review ensured >80% tumor cellularity prior to extraction.

### Whole-exome sequencing and analysis

Genomic DNA was extracted using the DNeasy & RNeasy kit (Qiagen). Exonic regions were captured with Agilent SureSelectXT Human All Exon V6 probes, and libraries were sequenced on the Illumina NovaSeq 6000 platform (150 bp paired-end reads). Median sequencing depth was ×300 for tumor tissues and PDOs (passage1), and ×100 for normal tissues. Quality control of raw reads was performed using FastQC (v0.11.9), Cutadapt (v2.5), and Trimmomatic (v0.39). Clean reads were aligned to the UCSC human reference genome (hg19) using bwa-mem2 (v2.0). BAM file processing—including sorting, merging, and indexing—was performed with Samtools (v1.10), and PCR duplicates were removed using GATK (v4.1.2.0). Coverage statistics were calculated using Samtools based on the Sure Select All ExonV6r2 BED file coordinates. Somatic point mutations and indels were called using GATK Mutect2 (v4.1.2.0) in paired tumor–normal mode. DNA were extracted from fresh-frozen tissues and passage 1 organoids. We also confirmed that histological review ensured >80% tumor cellularity prior to extraction.

### Phylogenetic tree construction

Phylogenetic relationships among multi-regional organoids were inferred as described previously (18). Genomic sequences (± 20 bp surrounding mutation sites) were extracted and used to construct phylogenetic trees with MEGA5 using the maximum parsimony algorithm. Driver mutations were annotated along the tree’s root, branches, and leaves to depict clonal evolution.

### Differential gene expression and gene set enrichment analysis

Differentially expressed genes (DEGs) between experimental groups were identified using DESeq2, with an absolute fold-change cutoff of> 2 and an adjusted P-value < 0.05. Comparisons were made between Invasive (T1/T2) *vs*. Superficial (T3/T4) for EMT analysis, and Sensitive (T2/T4) *vs*. Resistant (T1/T3) for 5-FU response. The ranked gene lists were used for gene set enrichment analysis (GSEA) with a previously published stem cell gene set and the GO_BP gene sets from MSigDB (v7.5.1). A q-value < 0.25 was considered statistically significant. KEGG and GO pathway enrichment analyses (19, 20)were conducted using the clusterProfiler R package.

(https://bioconductor.org/packages/release/bioc/html/clusterProfiler.html) We get permission to use the KEGG software from the Kanehisa laboratory.

### Statistical analysis

Statistical comparisons were performed using Student’s *t*-test in GraphPad Prism 10.0b. Data are presented as mean ± standard deviation (SD). A *P*-value < 0.05 was considered statistically significant.

## Results

### Case presentation

**Figure 1:**
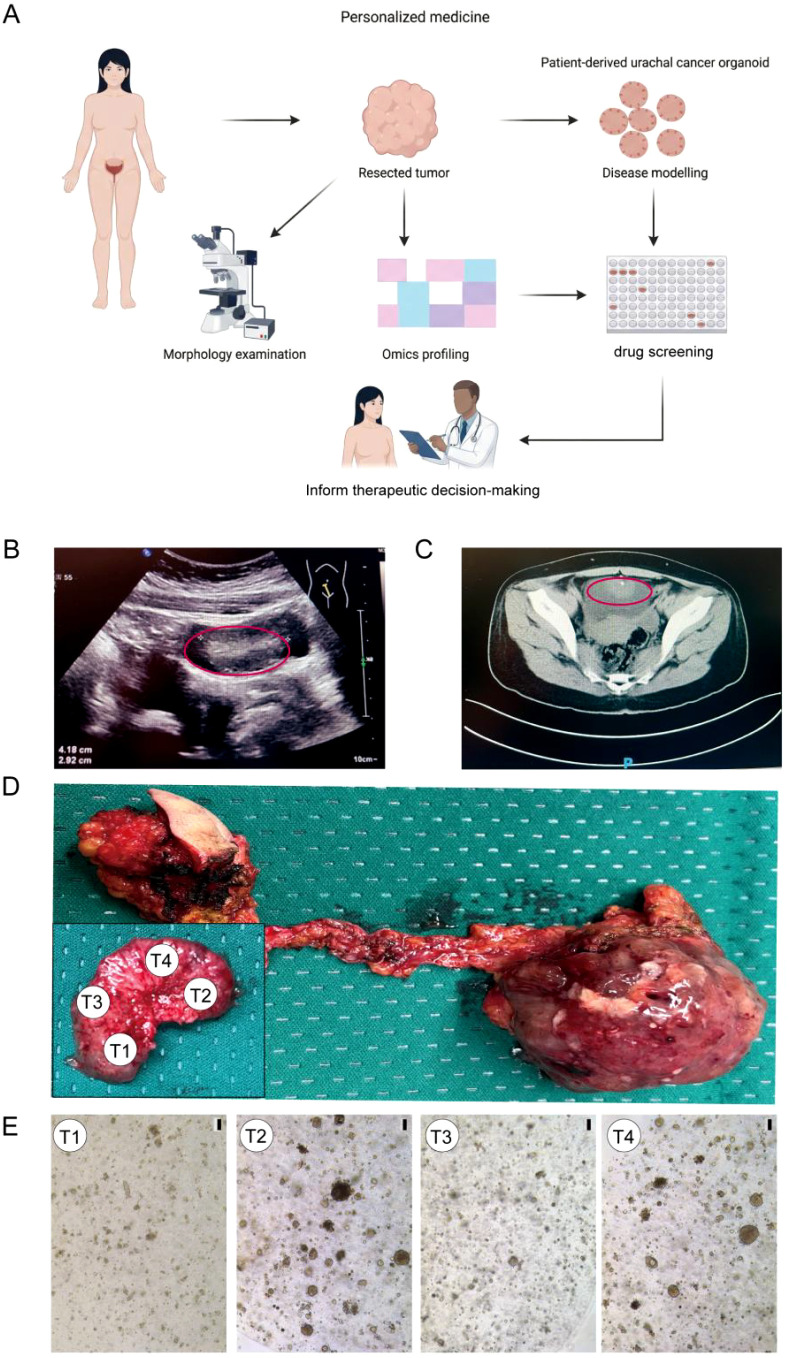
Establishment of multi-regional urachal carcinoma (UrC) patient-derived organoids. **(A)** A schematic overview for the generation and culturing of UrC organoids. **(B)** Ultrasonography revealed a protruding lesion measuring 3.2 × 3.1 × 3.5 cm on the anterior bladder wall. **(C)** Contrast-enhanced CT confirmed a solid anterior bladder wall mass of 4.2 × 2.9 cm. **(D)** Gross morphology of the surgically resected UrC specimen. **(E)** Bright-field images of patient-derived organoids (PDOs) passage 0, from spatially distinct tumor regions. Organoid: T1and T3 at day 5, Organoid:T2 and T4 at day 10.Scale bars: 100 μm.

A 31-year-old female presented in November 2023 for urological evaluation following six months of intermittent, painless gross hematuria accompanied by mucous discharge. Initial ultrasonography of the urinary tract identified a 3.2 × 3.1 × 3.5 cm hypoechoic nodule with indistinct margins along the anterior bladder wall. Cystoscopy confirmed the presence of a sessile, non-papillary lesion at the same anatomical site. Contrast-enhanced computed tomography (CT) further characterized a solid, calcified mass measuring 4.2 × 2.9 cm in the anterior bladder wall (**Figures 1B, C**). To exclude metastatic dissemination from gastrointestinal origins, a colonoscopy and biopsy were performed (**Supplementary Figure 1A**), which revealed a low-grade tubular adenoma (**Supplementary Figure 1B**), supporting the diagnosis of a primary urachal carcinoma (UrC) and excluding a metastasis from an intestinal tumor.

Intraoperative exploration revealed a broad-based, well-demarcated 3.2 × 3.1 cm mass on the anterior bladder wall, distinct from surrounding mucosa. Guided by cystoscopy, an en bloc resection was performed, which included the tumor, adjacent normal mucosa, partial umbilical tissue, urachal remnant, and adherent peritoneal structures (**Figure 1D**). Histopathological analysis confirmed the diagnosis of urachal adenocarcinoma.

### Establishment of multi-regional urachal carcinoma patient-derived organoids

To model intratumoral heterogeneity, fresh surgical specimens were subjected to immediate multi-regional sampling for PDO establishment and multi-omics analysis (**Figures 1A, E**). Four spatially distinct tumor fragments were cultured to generate organoids, T1 and T2 derived from histologically invasive regions, and T3 and T4 from morphologically superficial, non-invasive areas, which were confirmed by histopathological mapping. Using this approach, we have generated independent four urachal organoid lines. These lines have been propagated by serial passaging (up to passage 3) (**Supplementary Figure 2**).

### Urachal organoids retain histological, genomic, and transcriptomic features of parental tumors

**Figure 2:**
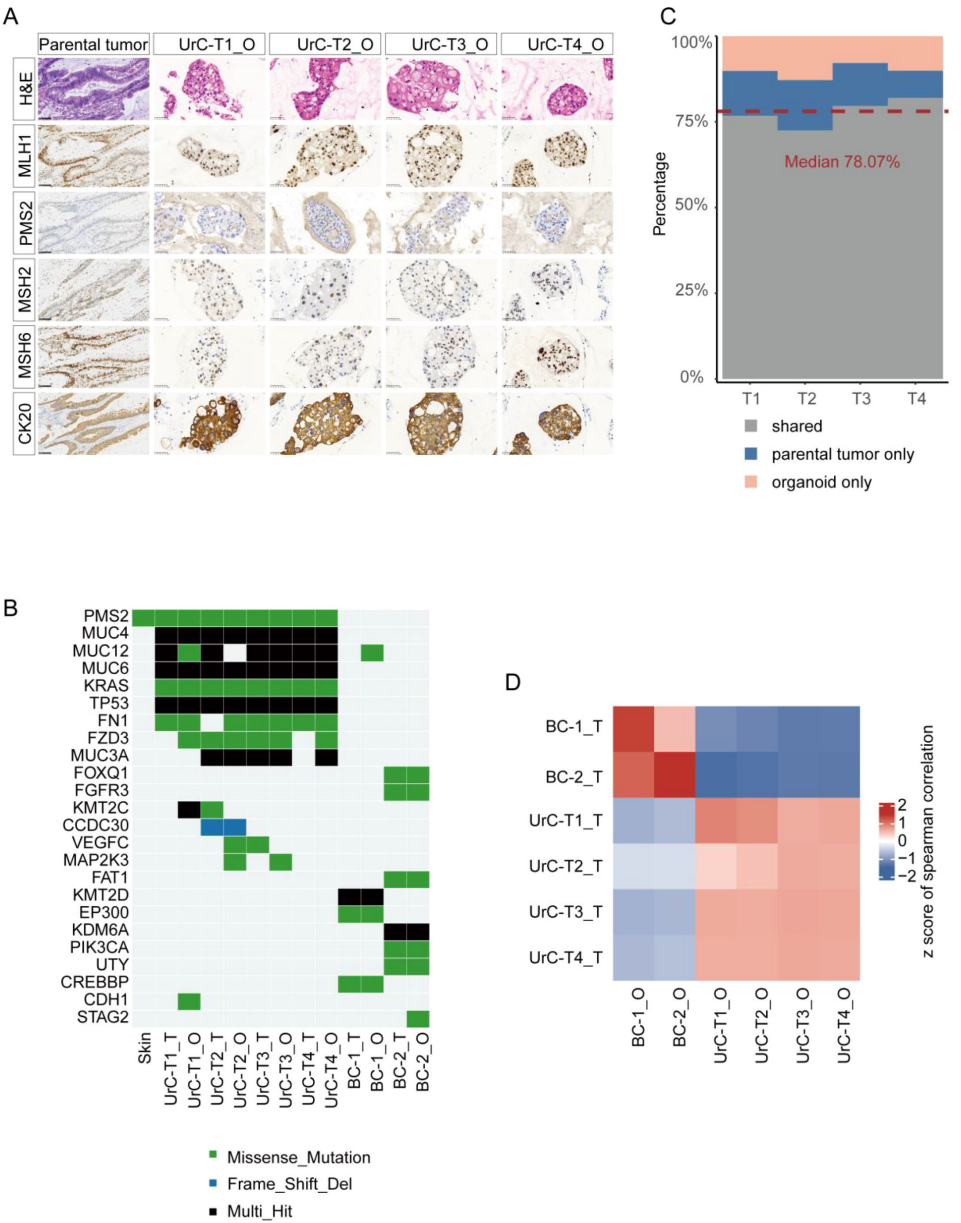
UrC organoids preserve the histological, genomic, and transcriptomic features of parental tumors. **(A)** Histological and immunohistochemical comparison of UrC organoids and matched tumor tissues, showing staining for mismatch repair proteins (PMS2, MLH1, MSH2, MSH6) and luminal marker CK20. Organoids: passage 1, Scale bars: 50 μm, 100 μm. **(B)** Whole-exome sequencing (WES) reveals mutational profiles of four UrC organoid-tumor pairs, with two bladder cancer (BC) organoids as controls. Organoids: passage 1. **(C)** Concordance analysis of cancer-related somatic variants between UrC tumors and their corresponding organoids. Median concordance is indicated. **(D)** Heatmap of Spearman correlation coefficients for transcriptomic profiles among UrC (*n* = 4), BC (*n* = 2) organoids, and parental tumors.

To assess fidelity between organoids and their parental tumors, we performed hematoxylin and eosin (H&E) staining and immunohistochemistry for canonical UrC markers, including cytokeratin 20 (CK20) and the mismatch repair (MMR) proteins, MSH2, MSH6, PMS2, and MLH1. As shown in **Figure 2A**, the organoids exhibited a histological architecture comparable to that of the parental tumor. Both the original tumor and derived PDOs demonstrated a mismatch repair-deficient (d-MMR) phenotype, evidenced by the selective loss of PMS2 expression alongside retention of MLH1, MSH2, and MSH6. Importantly, this d-MMR phenotype is rare in UrC, occurring in only approximately 3-4% of cases (21). PD-L1 expression rates are typically as low as 3.4% in unselected UrC patient cohorts (22).

Additionally, periodic acid–Schiff (PAS) staining confirmed mucin secretion capacity of the organoids (**Supplementary Figure 3**), consistent with their adenocarcinomatous identity. Thus, the established UrC PDOs faithfully mirrored both the histopathological and molecular features of the parental tumors.

Whole-exome sequencing (WES) was conducted on four UrC tumor-organoid pairs (median depth: 300×), two bladder cancer (BC) tumor-organoid pairs (median depth: 300×), and one matched normal skin sample as a somatic control (median depth: 100×). Detailed clinicopathological data for bladder cancer organoids are summarized in **Supplementary Table 1**.In line with previous genomic analyses of UrC (14, 23), recurrent mutations in *TP53* and *KRAS* were identified in all UrC samples (**Figure 2B**). Immunohistochemistry and WES consistently confirmed MMR deficiency due to a somatic missense mutation in *PMS2*. Notably, UrC tumors and organoids shared a median of 78.07% of somatic cancer-related variants (**Figure 2C**). In contrast, BC samples displayed distinct mutational signatures with a higher burden of alterations in epigenetic regulators, such as *EP300*, *KMT2D*, and *KDM6A* (**Figure 2B**).

Transcriptomic profiling via RNA-seq revealed high transcriptional concordance between tumor-organoid pairs (average Pearson’s *r* = 0.77). Hierarchical clustering demonstrated distinct clustering between UrC and BC samples, further supporting tumor-type specificity (**Figure 2D**). Collectively, these multi-omics data confirm that UrC PDOs faithfully recapitulate the genomic and transcriptional landscapes of their tumors of origin and provide a robust platform for modeling disease heterogeneity.

### Urachal carcinoma organoids recapitulate tumor heterogeneity

**Figure 3:**
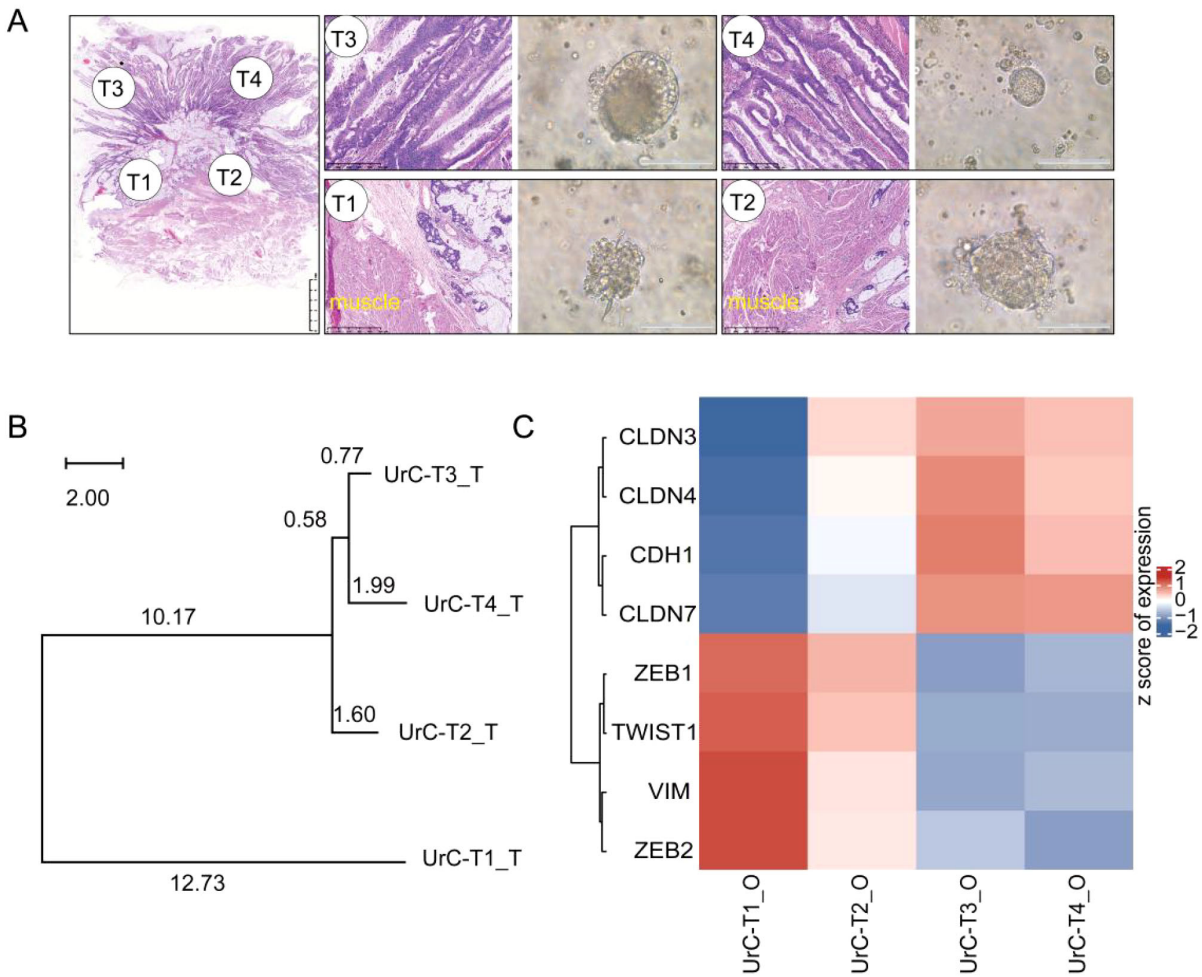
Urachal carcinoma organoids recapitulate intratumoral heterogeneity. **(A)** Histological analysis shows distinct morphologies in superficial (T3, T4) versus invasive (T1, T2) tumor regions: intestinal-type adenocarcinoma *vs*. mucinous adenocarcinoma, respectively. **(B)** Phylogenetic reconstruction of multi-regional UrC tissues based on WES. **(C)** RNA-seq-based heatmap of epithelial–mesenchymal transition (EMT) gene expression profiles across UrC organoids.

To investigate the organoids’ capacity to preserve intra-tumoral heterogeneity, we analyzed four PDOs generated from anatomically distinct regions of the tumor mass. Histological analysis of the primary tumor revealed pronounced heterogeneity, including superficial intestinal-type adenocarcinoma of moderate differentiation, including areas of mucinous adenocarcinoma with invasion into the bladder wall muscularis propria (**Figure 3A**). Phylogenetic tree reconstruction based on genomic data demonstrated both branching and parallel evolutionary patterns among the sampled tumor regions (**Figure 3B**).

Morphologically, PDOs also reflected their site of origin. Organoids T1 and T2, derived from invasive regions, displayed irregular contours with stellate projections—features previously associated with aggressive phenotypes and epithelial-mesenchymal transition (EMT) (24, 25). In contrast, T3 and T4 organoids from superficial areas retained smooth borders. To evaluate EMT status, RNA-seq analysis compared gene expression profiles between T1/T2 (invasive) and T3/T4 (superficial) PDOs. Mesenchymal markers (*TWIST1*, *VIM*) were enriched in T1/T2, while epithelial markers (*CLDN3*, *CDH1*, *CLDN4*) were upregulated in T3/T4 (**Figure 3C**). Differential gene expression and pathway enrichment analyses further identified activation of EMT-related pathways in T1/T2 organoids, including ECM-receptor interaction (26–28), Wnt/β-catenin, and PI3K-AKT-mTOR signaling (**Supplementary Figure 4A**). Gene Set Enrichment Analysis (GSEA) validated significant Wnt pathway activation in the invasive PDOs (**Supplementary Figure 4B**).

Together, these findings demonstrate that UrC PDOs not only recapitulate the molecular and morphological diversity of the parental tumor but also maintain regional differences in EMT-associated signaling and invasive potential, reinforcing their value for studying intratumoral heterogeneity.

### Screening of clinically relevant agents in urachal carcinoma organoids reflects patient response

**Figure 4:**
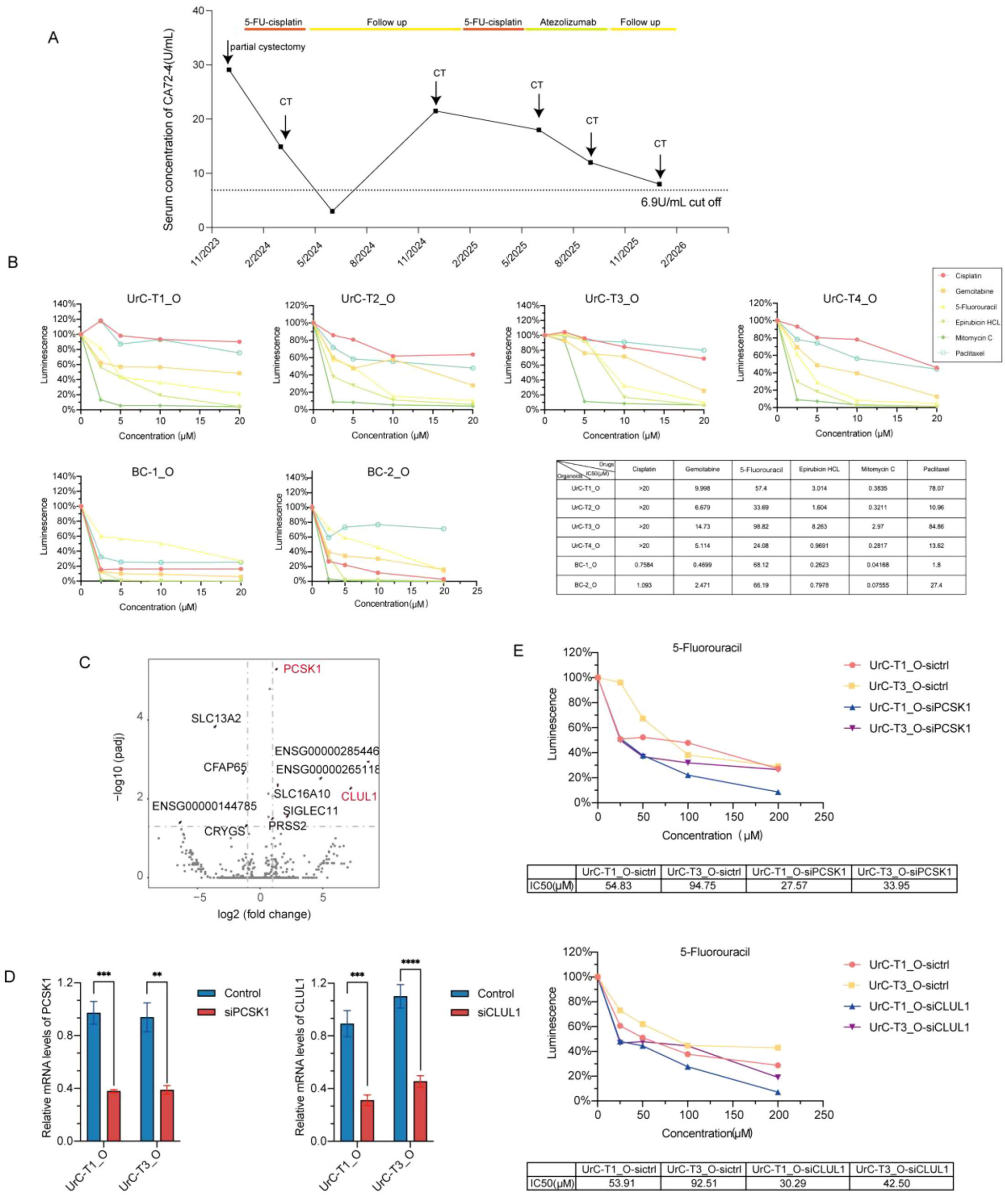
UrC organoids predict patient response to clinically relevant chemotherapeutics. **(A)**Timeline of serum CA72–4 serum marker levels with surgical and systemic treatments and imaging follow-up examinations. **(B)** Dose–response curves for 5-FU, cisplatin, and other drugs in four UrC and two BC organoids. Viability was measured after 6 days using CellTiter-Glo. **(C)** Volcano plot of DEGs distinguishing 5-FU-sensitive (T2, T4) *vs*. 5-FU-resistant (T1, T3) UrC organoids. **(D, E)** siRNA-mediated knockdown of *PCSK1* and *CLUL1* sensitized T1 and T3 organoids to 5-FU. Data are presented as mean ± SD (*n* = 3). **P < 0.01, ***P < 0.001,****P < 0.0001, two-tailed Student’s *t*-test.

The patient initially underwent partial cystectomy with en bloc resection of the urachal ligament and umbilicus in November 2023, subsequently receiving adjuvant 5-fluorouracil (5-FU)-based chemotherapy from December 2023 to February 2024. Disease progression was later documented on follow-up computed tomography (CT), coinciding with a marked elevation in serum CA72–4 levels. In contrast, CA19-9 (8.33 IU/mL), CA242 (2.55 IU/mL), and CEA (0.47 μg/L) remained within normal limits throughout this period. This biochemical and radiographic progression prompted the initiation of immunotherapy in May 2025, leading to a partial response by August 2025. Follow-up continued through December 2025 (**Figure 4A**).

To evaluate the predictive capacity of patient-derived organoids (PDOs) in therapeutic response, we screened the four UrC PDOs established from surgical tumor specimens and two bladder cancer (BC) PDOs against six commonly used chemotherapeutic agents, including 5-FU and cisplatin. As shown in **Figure 4B**, while BC PDOs were sensitive to cisplatin, all four UrC PDOs demonstrated complete resistance. Notably, two UrC PDOs (T2 and T4) displayed relative sensitivity to 5-FU, exhibiting IC_50_ values below the predefined efficacy threshold of 50 μM (29, 30). However, the remaining two PDOs were resistant. Considering that therapeutic resistance is often driven by the most refractory subclones within a tumor, our findings suggest an overall chemoresistant phenotype in this case, aligning with the patient’s clinical outcome (**Figure 4A**). These results underscore the utility of UrC PDOs in accurately reflecting patient-specific drug responses.

To further explore the molecular underpinnings of 5-FU response, we conducted transcriptomic profiling comparing the 5-FU-sensitive and -resistant UrC PDOs. As shown in **Figure 4C**, *PCSK1* and *CLUL1* were significantly upregulated in the resistant group. Importantly, targeted knockdown of *PCSK1* or *CLUL1* in T1 and T3 PDOs, respectively, resulted in increased sensitivity to 5-FU treatment (**Figures 4D, E**). These findings suggest a functional role for both genes in modulating chemoresistance of UrC to 5-FU treatment. Of particular note, *PCSK1* and *CLUL1* overexpression have previously been associated with poor prognosis and therapeutic resistance in rectal adenocarcinoma (31), indicating a potentially conserved mechanism across epithelial malignancies. Furthermore, our validation analysis using The Cancer Genome Atlas (TCGA) colorectal cancer dataset-selected due to its molecular similarity to UrC-confirmed that overexpression of PCSK1 and CLUL1 correlates with therapeutic resistance of 5-FU in larger patient cohorts(**Supplementary Figure 5**). These findings support the potential relevance of these genes as biomarkers, notwithstanding the limited sample size of our initial cohort. Altogether, these results identify candidate biomarkers predictive of 5-FU response and offer a framework for the mechanistic dissection of chemoresistance in UrC.

### Drug response profiling of Urachal carcinoma organoids

**Figure 5:**
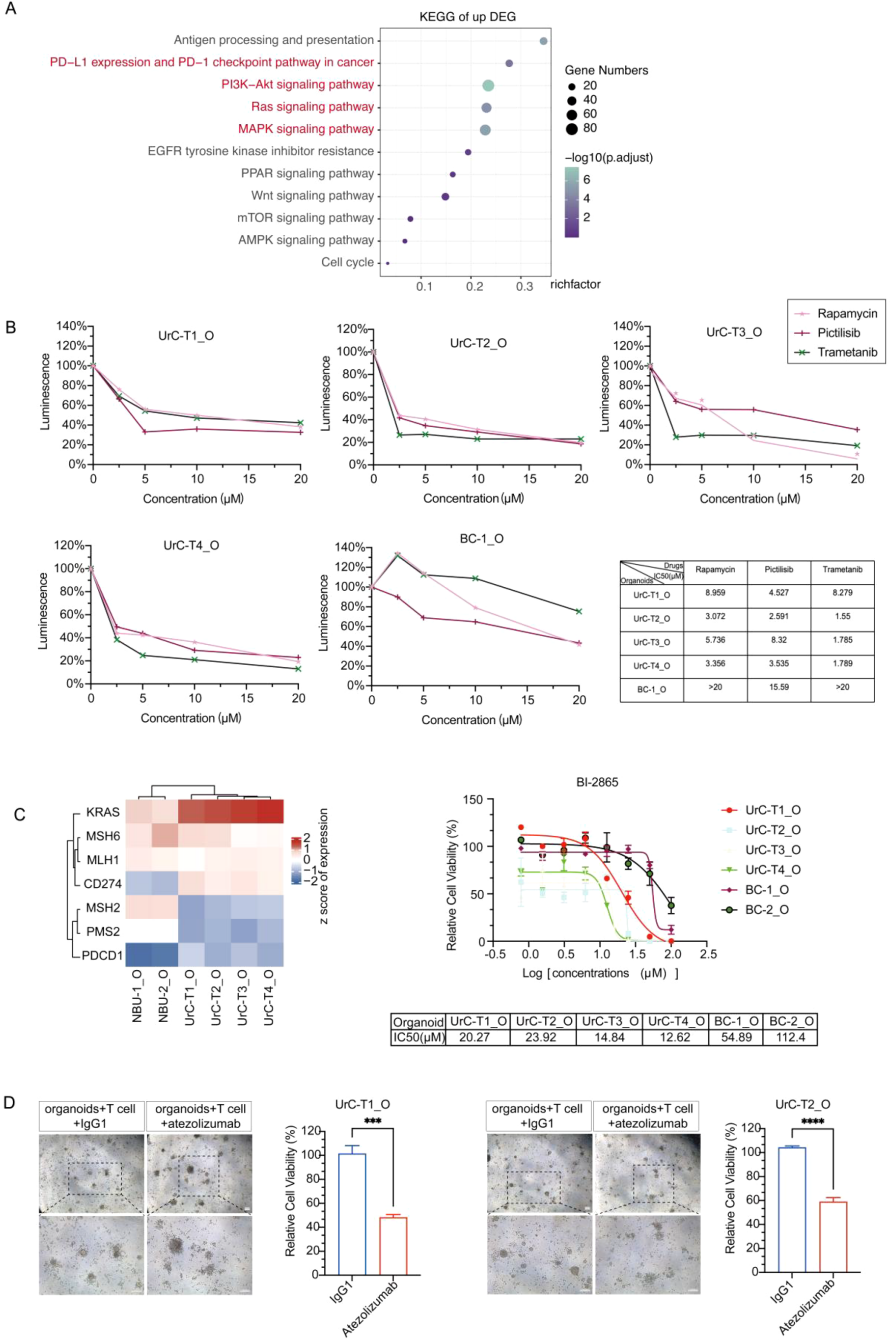
Molecular profiling reveals potential therapeutic targets in UrC. **(A)** KEGG (www.kegg.jp/kegg/kegg1.html) enrichment analysis of DEGs between four UrC organoids and two normal urothelial organoids. **(B)** Dose–response curves of selected inhibitors (targeting PI3K, mTOR, MEK) tested in UrC and BC organoids. Viability normalized to DMSO controls. Data represent means ± SD from triplicates. **(C)** Left: RNA-seq-derived heatmap showing expression of candidate therapeutic targets (e.g., *KRAS*, *PD-L1*) in UrC organoids. Right: Dose–response curves of KRAS inhibitor BI-2865 in UrC and BC organoids. **(D)** PD-L1 blockade enhances cytotoxicity in UrC organoids. T cell co-cultures treated with 20 μg/mL atezolizumab or isotype control were assessed for viability at 72 hours. Error bars: mean ± SD, ***P < 0.001, ****P < 0.0001; *t*-test, respectively.

Given the patient’s resistance to standard chemotherapy (**Figure 4**), we next assessed the efficacy of targeted therapies based on insights from whole-exome and transcriptomic profiling. Comparative transcriptomic analysis between two normal bladder urothelial PDOs and four UrC PDOs revealed significant dysregulation in several oncogenic pathways in UrC, including PD-L1/PD-1 checkpoint signaling, Ras signaling, and MAPK cascades (**Figure 5A**). Functional drug screening demonstrated that UrC PDOs were significantly more sensitive than a BC PDO (lacking MAPK-activating mutations such as *PIK3CA* or *FGFR3*) to the PI3K inhibitor pictilisib, the mTOR inhibitor rapamycin, and the MEK inhibitor trametinib (**Figure 5B**). These responses strongly correlated with the transcriptional profiles of the PDOs, highlighting the mechanistic relevance of pathway activation to therapeutic vulnerability.

Moreover, whole-exome sequencing revealed *KRAS* and *PMS2* mutations in the UrC PDOs (**Figure 2B**), and transcriptomic data confirmed elevated expression of *KRAS* and *PD-L1* (**Figure 5C**), supporting their potential as therapeutic targets. We therefore evaluated the efficacy of the KRAS inhibitor BI-2865 and the PD-L1 inhibitor atezolizumab. Dose-response assays showed that the IC_50_ values of UrC PDOs to BI-2865 ranged from 12.62 μM to 23.92 μM, markedly lower than those of BC PDOs (54.89 μM to 112.4 μM), indicating enhanced KRAS inhibitor sensitivity in UrC organoids (**Figure 5C**). Furthermore, treatment with atezolizumab led to significant cytotoxic responses in UrC PDOs, with cell viability decreased by 53.42% ± 4.11% and 45.28% ± 2.19% in T1 and T2 PDOs, respectively (**Figure 5D**).

Collectively, these results demonstrate that molecular profiling of UrC PDOs enables the identification of alternative, actionable therapeutic targets. This approach supports the integration of PDO-based drug screening into personalized treatment strategies for rare malignancies such as urachal carcinoma.

## Discussion

Systemic therapy is frequently required in urachal carcinoma (UrC) due to its late-stage presentation or progression following locoregional interventions. However, treatment options remain limited. Chemotherapy efficacy data are largely confined to retrospective studies, as the rarity of UrC precludes prospective clinical trials. Current treatment strategies rely on empirical use of conventional chemotherapies, which often yield suboptimal outcomes. Moreover, evidence supporting targeted therapies is scarce, and no validated alternatives exist once resistance develops. Therefore, there is a pressing need for reliable experimental models to address the clinical challenges unique to UrC.

In this study, we successfully established four UrC patient-derived organoid (PDO) lines from a 31-year-old female patient carrying somatic *PMS2* mutations. Histological and immunohistochemical analyses confirmed that the PDOs preserved key features of the parental tumors, including mucin production and expression of canonical markers such as CK20 and MLH1 (**Figure 2**). Genomic and transcriptomic profiling further demonstrated high fidelity between organoids and corresponding tumor tissues (**Figure 2**), supporting the robustness of these models for translational applications. PDO platforms represent a transformative tool in precision oncology, enabling the integration of mutational and transcriptomic data to identify therapeutic vulnerabilities. While previous studies have applied drug screening approaches in UrC ex vivo models to identify potential active agents (32, 33), the mechanistic basis linking drug response to specific genomic alterations has remained underexplored. Our PDO-based platform addresses this gap by integrating functional and molecular profiling. In our study, differentially expressed gene (DEG) analysis and KEGG pathway enrichment revealed activation of the RAS/MAPK, PI3K/AKT/mTOR, and PD-L1 signaling pathways as potential therapeutic targets (**Figure 5A**). Whole-exome sequencing identified a *KRAS^G12V^* driver mutation, consistent with constitutive activation of MAPK signaling (34), providing a mechanistic basis for sensitivity to pathway-specific inhibitors. Functional validation using targeted therapies confirmed that drug responses were in strong concordance with molecular profiles (**Figure 5B**), highlighting the predictive value of PDO-based pharmacotyping. Intratumoral heterogeneity (ITH) presents a major barrier to achieving durable therapeutic responses (35). Our multi-regional sampling approach captured spatial heterogeneity within the tumor, reflected in differential drug sensitivity across UrC PDOs (**Figure 4**). While dependency on the MAPK pathway was predominant, subpopulations exhibiting resistance via pathway-independent mechanisms were also observed (**Figure 5**). These findings emphasize the importance of integrating spatially resolved tumor sampling with PDO pharmacotyping to inform more comprehensive and effective therapeutic strategies.

Immune checkpoint blockade targeting PD-1/PD-L1 is emerging as a promising therapeutic approach, particularly in tumors with microsatellite instability (MSI) status (36).The PDOs in our study exhibited dMMR/MSI features, including loss of PMS2 and elevated PD-L1 expression. Mechanistically, MSI tumors accumulate neoantigens that enhance immunogenicity and sensitize tumors to immune checkpoint inhibitors (37–39). Consistent with this, *in vitro* drug assays demonstrated that PD-L1 blockade via atezolizumab augmented T cell-mediated cytotoxicity. These results align with recent clinical reports, including a case of stable disease in a UrC patient treated with atezolizumab (23), and Consistent with the established efficacy of PD-1/PD-L1 inhibitors in microsatellite instability-high (MSI-H) malignancies, Jia et al. reported that two MSI-H (MSH6-loss, PD-L1 TPS≥1%) urachal carcinoma patients treated with nivolumab achieved stable disease (40). However, it is critical to emphasize that UrC is predominantly an “immune cold” tumor with PD-L1 expression rates as low as 3.4% in unselected cohorts (22).Multiple studies have documented limited efficacy of immune checkpoint inhibitors in unselected UrC cohorts lacking MSI-high features (33, 41).This case should not be generalized to suggest that all UrC patients would benefit from PD-L1 blockade. Rather, immune checkpoint inhibitors should be considered specifically in patients with confirmed MSI-high/d-MMR status and in those with high PD-L1 expression observed upon histochemical characterization. Overall, our data underscore the potential of combining histological, genomic, and transcriptomic insights with functional drug screening to advance precision medicine in UrC.

This study has several limitations. First, the small sample size reflects the rarity of UrC, which limits the statistical power and generalizability of the findings. Second, current PDO models do not incorporate immune components, limiting their utility for evaluating immunotherapy. The development of tumor–immune co-culture systems will be crucial to overcome this limitation. Ultimately, to address these challenges, we are launching a longitudinal biospecimen collection and functional validation pipeline to extend this research and enable future clinical implementation.

From a practical clinical implementation perspective, we acknowledge that the cost, logistical complexity, and time requirements of establishing multiple PDO lines from multi-regional sampling present significant barriers to routine first-line use. Therefore, we propose a sequential workflow that leverages the complementary strengths of molecular profiling and functional testing. Next-generation sequencing should serve as the first-line tool for initial molecular characterization and identification of actionable targets, as it is more rapid, economical, and widely accessible. Patient-derived organoids would be most valuable in later stages of treatment, particularly when patients develop therapeutic resistance. In such scenarios, PDOs can be established from specific refractory lesions to functionally test salvage therapies and identify resistance mechanisms driven by intratumoral heterogeneity. Alternatively, we may need to consider the patient’s preferences alongside other factors when deciding between NGS and PDO.

## Conclusions

In summary, d-MMR/MSI UrC PDOs reproduce the phenotypic and molecular features of their parental tumors, capturing intratumoral heterogeneity. Thus, they serve as a valuable platform for reflecting treatment responses, investigating resistance mechanisms, and developing personalized therapeutic regimens.

## Data Availability

The datasets presented in this study can be found in online repositories. The names of the repository/repositories and accession number(s) can be found in the article/**Supplementary Material**.

## Supplementary material

Supplementary Figure 1 Clinical assessment of the UrC patient. **(A)** Colonoscopy revealed a 0.5 cm flat polypoid lesion. **(B)** Biopsy of the lesion showed a low-grade tubular adenoma (H&E staining).

Supplementary Figure 2 Passage of UrC organoids. Bright-field of representative passaged organoids.Scale bar: 100 μm.

Supplementary Figure 3 Mucin production in UrC organoids. Periodic acid–Schiff (PAS) staining of UrC organoids. Bright-field (left) and PAS-stained (right) images highlight intracellular mucin (red arrows) and extracellular mucin (blue arrows). Organoid: passage 2 at day 21.Scale bar: 100 μm.

Supplementary Figure 4 Pathway enrichment and gene set enrichment analyses (GSEA). **(A)** Pathway (http://www.kegg.jp/kegg/kegg1.html) enrichment analysis of DEGs between invasive (T1, T2) and superficial (T3, T4) UrC organoids. **(B)** GSEA indicates activation of Wnt/β-catenin signaling in T1/2 compared to T3/4 organoids.

Supplementary Figure 5 *PCSK1* and *CLUL1* levels are correlated with 5-FU sensitivity. The correlation between PCSK1/CLUL1 gene expression and sensitivity to 5-fluorouracil (5-FU) was assessed using Spearman’s rank correlation coefficient. The distribution of 5-FU sensitivity data is visualized on the right density curve, while the upper density curve represents the distribution of gene expression levels.

Supplementary Table 1 Clinicopathological features of patient-derived bladder tumor organoid.

Supplementary Table 2 Sequences of the DNA primers for qRT-PCR.
