# Supplementary material for: Patient-derived d-MMR/MSI phenotype urachal cancer organoids for personalized drug screening
Source: Front Oncol. 2026 Mar 5;16:1773072. doi: 10.3389/fonc.2026.1773072 (PMC12999449; doi:10.3389/fonc.2026.1773072)
Supplement: Supplementary file 1 [file Table1.docx]

Supplementary tables

Table S1. Clinicopathological features of patient-derived bladder tumor organoid

| Line | Overall tumor stage | Pathologic classification of parental tumor sample | Sex | Age | Smoking status | Prior intravesical therapy | Prior  systemic  therapy | Tumor volume |
| --- | --- | --- | --- | --- | --- | --- | --- | --- |
| BC-1_O | T1 | Invasive high-grade papillaryurothelial carcinoma | Female | 71 | None | None | None | 4.9*2.7cm |
| BC-2_O | Ta | High-grade papillary urothelial carcinoma, non-invasive | male | 61 | Active | None | None | 2.0*1.3cm |

Table S2.Sequences of the DNA primers for qRT-PCR

| PCSK1(Human) | Forward | 5’-AATGAATGGGCAGCGGAGAT-3’ |
| --- | --- | --- |
|  | Reverse | 5’-TCAAGTGAACCAATCTGACCCA-3’ |
| CLUL1(Human) | Forward | 5’-AGATGACCCGGAAGCACTTG-3’ |
|  | Reverse | 5’-CAGACACCCAGCCAAATTGC-3’ |
