# Supplementary material for: Patient-derived d-MMR/MSI phenotype urachal cancer organoids for personalized drug screening
Source: Front Oncol. 2026 Mar 5;16:1773072. doi: 10.3389/fonc.2026.1773072 (PMC12999449; doi:10.3389/fonc.2026.1773072)

Supplementary Material

Supplementary Figures

**Figure S1. Clinical assessment of the UrC patient.**

1. Colonoscopy revealed a 0.5 cm flat polypoid lesion.
   (B) Biopsy of the lesion showed a low-grade tubular adenoma (H&E staining).

**Figure S2. Passage of UrC organoids.**

Bright-field of representative passaged organoids.Scale bar: 100 μm.

**Figure S3. Mucin production in UrC organoids.**

Periodic acid–Schiff (PAS) staining of UrC organoids. Bright-field (left) and PAS-stained (right) images highlight intracellular mucin (red arrows) and extracellular mucin (blue arrows). Organoid: passage 2 at day 21.Scale bar: 100 μm.

**Figure S4. Pathway enrichment and gene set enrichment analyses (GSEA).**

(A) Pathway (www.kegg.jp/kegg/kegg1.html) enrichment analysis of DEGs between invasive (T1, T2) and superficial (T3, T4) UrC organoids.

(B) GSEA indicates activation of Wnt/β-catenin signaling in T1/2 compared to T3/4 organoids.

**Figure S5.  *PCSK1* and *CLUL1* levels are correlated with 5-FU sensitivity.**

The correlation between PCSK1/CLUL1 gene expression and sensitivity to 5-fluorouracil (5-FU) was assessed using Spearman's rank correlation coefficient. The distribution of 5-FU sensitivity data is visualized on the right density curve, while the upper density curve represents the distribution of gene expression levels.

**Figure S1**

**
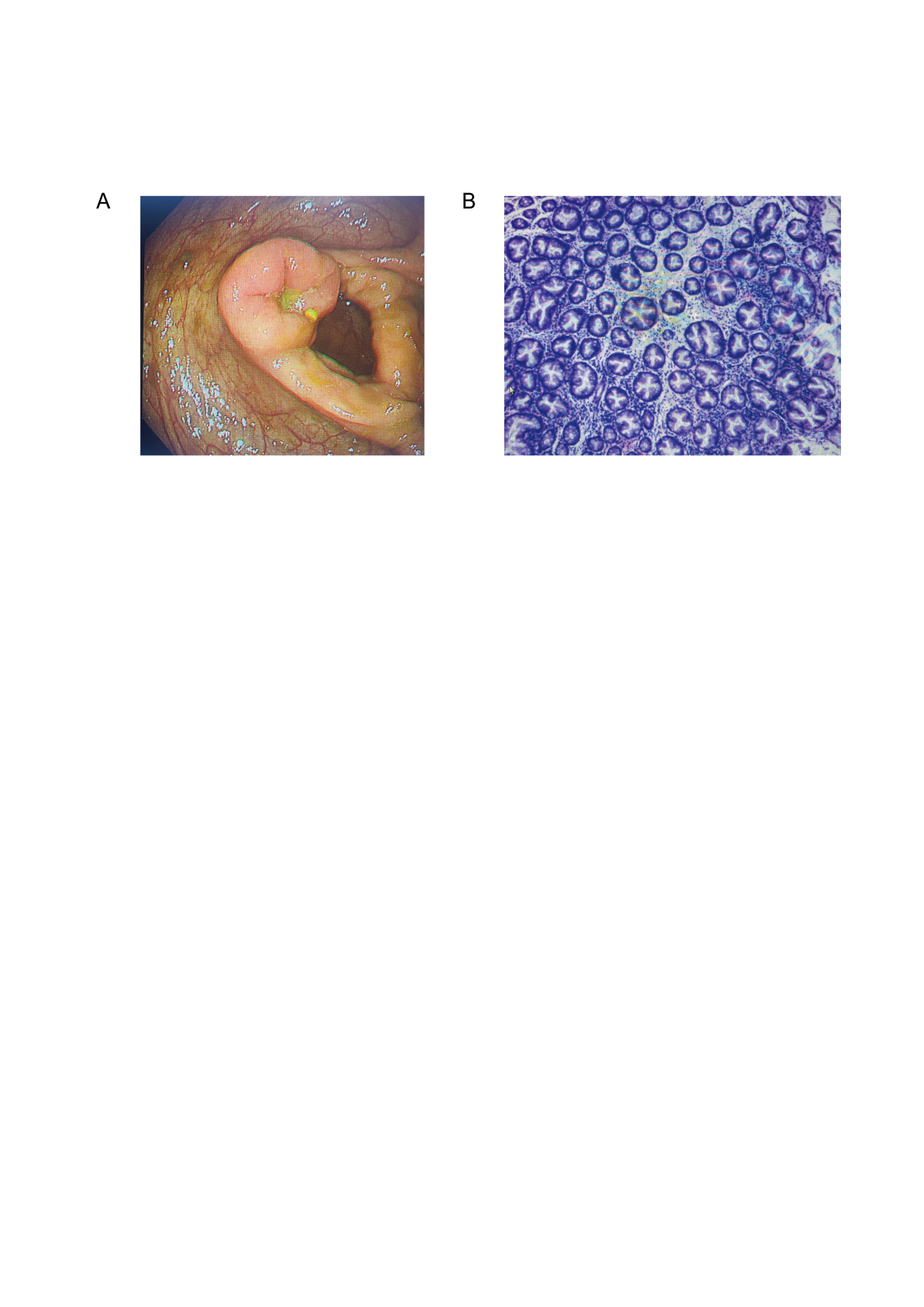
**

**Figure S2**

**
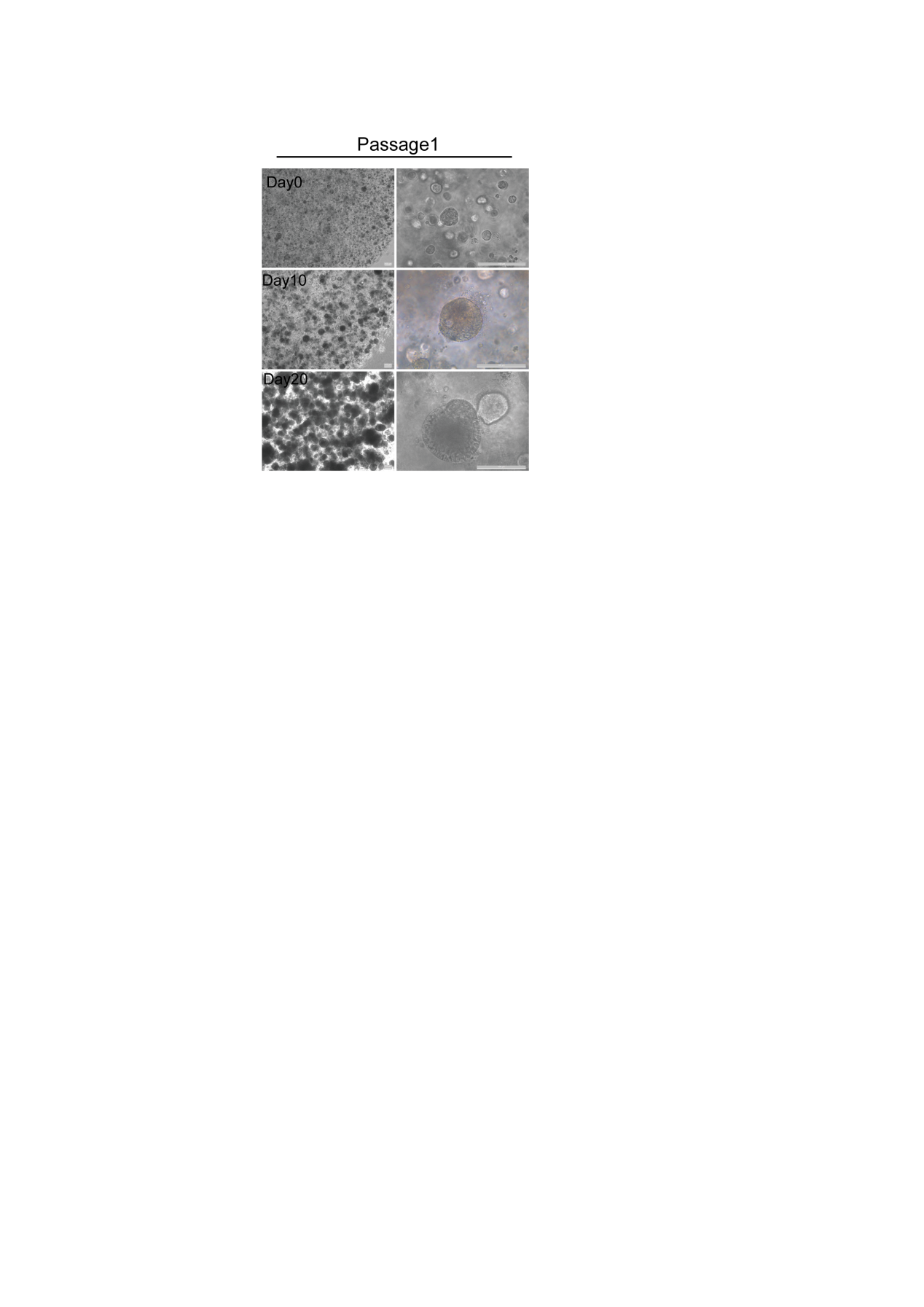
**

**Figure S3**


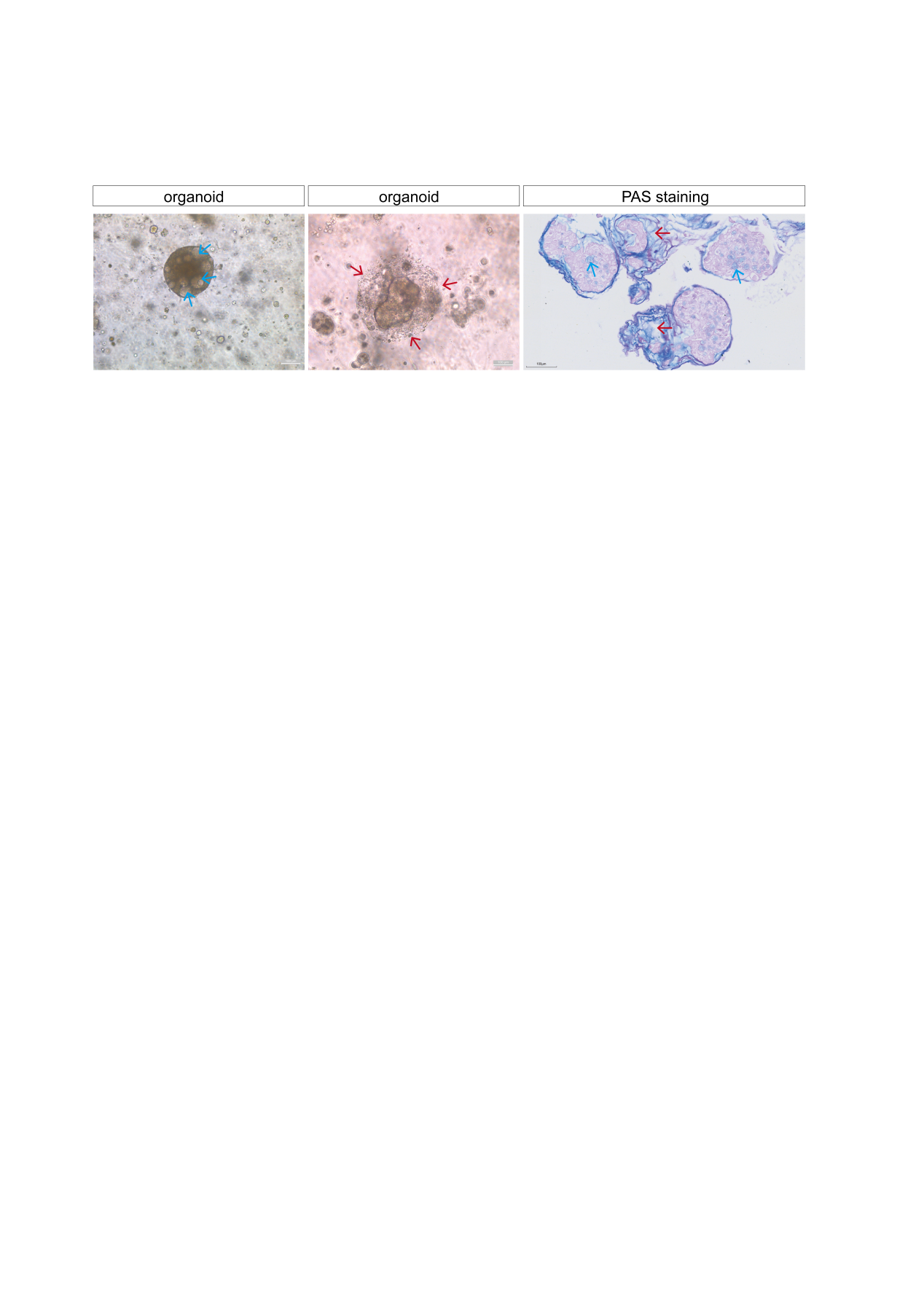


**Figure S4**


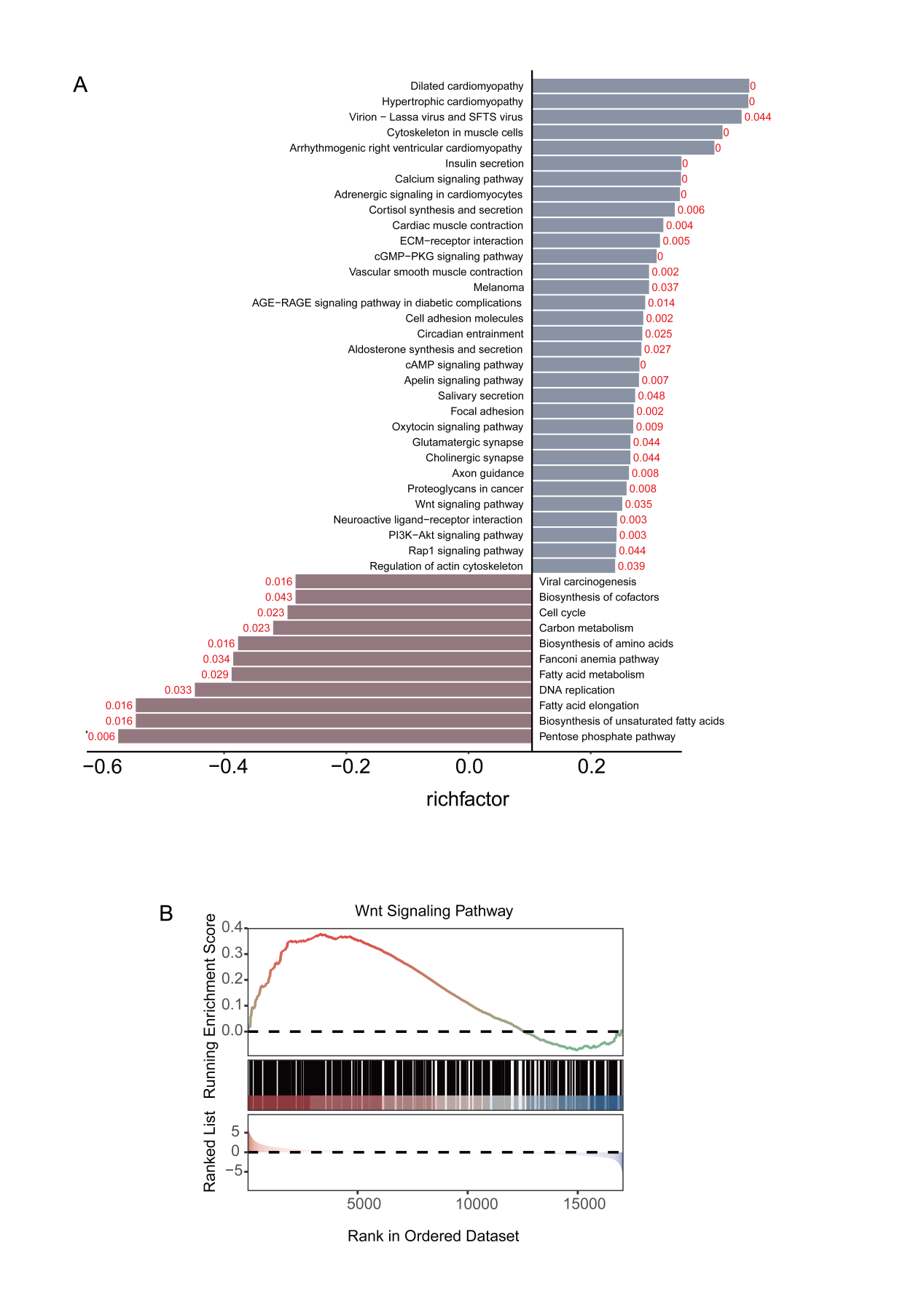


**Figure S5**


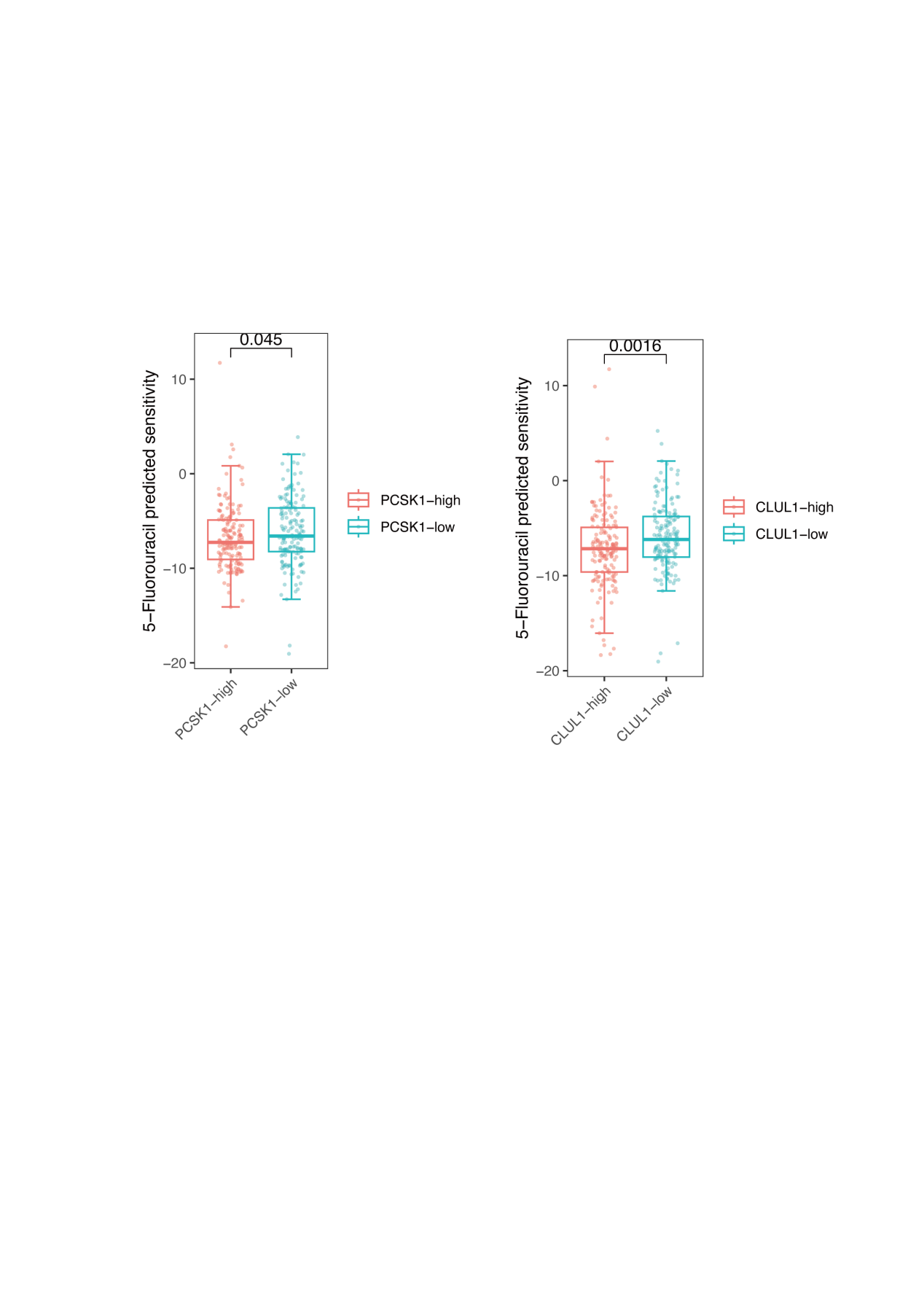

Supplement: Supplementary Figure 1 — Clinical assessment of the UrC patient. (A) Colonoscopy revealed a 0.5 cm flat polypoid lesion. (B) Biopsy of the lesion showed a low-grade tubular adenoma (H&E staining). [file DataSheet1.docx]
